# Perceived Time Spent on TikTok, Overall User Satisfaction, and Parallel Psychological Costs

**DOI:** 10.3390/bs16050816

**Published:** 2026-05-19

**Authors:** Qian Zhang, Jingjing Yang, Dongyoup Kim

**Affiliations:** College of Business Administration, Gachon University, Seongnam-daero, Sujeong-gu, Seongnam-si 13120, Gyeonggi-do, Republic of Korea; zq0924@gachon.ac.kr (Q.Z.); 201945088@gachon.ac.kr (J.Y.)

**Keywords:** social media, short video use, usage time perception, user satisfaction, psychological costs

## Abstract

With the rapid growth of short-video platforms, it has become increasingly important to understand the psychological processes that sustain prolonged engagement and contribute to individual evaluative responses. This study examines the dual pattern of associations involving perceived time spent on TikTok by investigating whether it is positively associated with overall user satisfaction while also being linked to psychological cost-related responses, including privacy concerns, health consciousness, social interaction anxiety, and social media fatigue. Data were collected through an online survey administered via Prolific and analyzed using structural equation modeling (SEM) and fuzzy-set qualitative comparative analysis (fsQCA). The findings show that perceived time spent on TikTok is significantly associated with health consciousness and social interaction anxiety. Perceived time spent on TikTok is also directly and positively associated with overall user satisfaction. Moreover, privacy concerns and social media fatigue are negatively associated with overall user satisfaction. The fsQCA results further reveal six configurations associated with high user satisfaction. These configurations illustrate the principle of equifinality and indicate that no single condition reached the conventional threshold for necessity. Overall, the findings suggest that high user satisfaction can coexist with different combinations of psychological cost-related responses, thereby offering a more nuanced account of how users experience short-video platforms.

## 1. Introduction

With the rapid expansion of the Internet in recent years, short-video content has grown dramatically (R. Wang et al., 2026). Among short-video platforms, TikTok has emerged as one of the fastest-growing platforms worldwide, surpassing 1.1 billion monthly active users since its international launch in 2018 (Ming et al., 2024). Its algorithmically driven content delivery, personalized recommendations, interactive features, and immersive scrolling interface have substantially altered how individuals consume entertainment and engage with social media (Jiang et al., 2025). These platform characteristics also foster a strong sense of prolonged engagement with TikTok (R. Wang et al., 2026). As daily usage continues to increase, social media has become not only a major source of entertainment but also an integral part of everyday social experience (H.-M. Wang et al., 2026).

At the same time, the characteristics that make TikTok engaging may also be linked to psychological cost-related responses. Its recommendation algorithm, abundant content supply, and continuous scrolling interface can encourage prolonged and sometimes difficult-to-regulate use (Qiao et al., 2024; Golding et al., 2025). Prior research also suggests that intensive short-video or social media use may be associated with emotional exhaustion and other adverse psychological experiences (Huang et al., 2023). These observations point to a central tension in TikTok use: perceived time spent on the platform may reflect enjoyable and engaging use, thereby enhancing overall user satisfaction, while simultaneously making users more aware of privacy-, health-, social-, and fatigue-related psychological costs. This tension raises an important question about how perceived time spent on TikTok is associated with both overall user satisfaction and psychological cost-related responses.

Previous research on social media has devoted considerable attention to user engagement (Xiao et al., 2023; Z. Zhang et al., 2025), content virality (Chu et al., 2024), and platform addiction (Covelli et al., 2025; Ozimek et al., 2025). By contrast, less is known about how perceived time spent on TikTok is associated with users’ psychological cost-related responses to platform use and how these responses coexist with overall user satisfaction. This issue is particularly relevant because time spent on engaging activities is often subjectively perceived rather than accurately recalled (Jiang et al., 2025; Tobin & Grondin, 2009). In the context of short-video consumption, perceived time spent may therefore capture users’ subjective sense of intensive platform engagement. This construct provides a useful basis for examining how TikTok use can be associated with both overall user satisfaction and psychological cost-related responses.

Moreover, most prior studies have treated user satisfaction as a relatively direct outcome of perceived usefulness or hedonic enjoyment (Hu & Lee, 2026; Pang & Zhang, 2024; Pereira & Tam, 2021), while paying less attention to whether perceived time spent can be associated with both satisfaction-enhancing engagement and psychological cost-related responses. This issue is particularly important for TikTok because its algorithmically curated recommendations, stimulating content, and continuous scrolling interface may heighten users’ subjective sense of engagement while making privacy-, health-, social-, and fatigue-related costs more salient.

To address this gap, the present study examines how perceived time spent on TikTok is associated with both overall user satisfaction and psychological cost-related responses. Specifically, we investigate whether perceived time spent is associated with overall user satisfaction while also linked to psychological cost-related responses, including privacy concerns, health consciousness, social interaction anxiety, and social media fatigue. We conceptualize perceived time spent on TikTok as a psychologically meaningful indicator of users’ subjective platform engagement, which may capture both satisfaction-enhancing experiences and perceived psychological costs. Accordingly, this study addresses the following research questions.

(1) How does perceived time spent on TikTok relate to psychological cost-related responses associated with social media use?
(2) How does perceived time spent on TikTok relate to overall user satisfaction?
(3) How do psychological cost-related responses relate to overall user satisfaction?

By conceptualizing perceived time spent on TikTok as a psychologically meaningful indicator of users’ subjective platform engagement, this study examines how it is associated with both overall user satisfaction and psychological cost-related responses. The findings provide a more nuanced account of short-video platform use by showing how perceived time spent may be linked to satisfaction-enhancing engagement while also coexisting with privacy-, health-, social-, and fatigue-related psychological costs.

## 2. Literature Review and Hypotheses Development

### 2.1. Perceived Time Spent on TikTok and Psychological Cost-Related Responses

#### 2.1.1. Perceived Time Spent on TikTok and Privacy Concerns

Privacy concerns refer to users’ apprehension about the collection, storage, and potential misuse of their personal information by digital platforms (Hong et al., 2025; H. Xu & Li, 2026). TikTok customizes its content feed through an algorithm that tracks users’ interaction patterns and video preferences, including watch time, likes, comments, shares, and follows, to provide personalized recommendations (Kang & Lou, 2022). As users perceive that they spend more time on TikTok, they may also become more aware that their platform activities generate behavioral data, which can heighten concerns about privacy risks.

Perceived time spent on TikTok may therefore make privacy-related costs more salient. The highly personalized nature of TikTok’s algorithm can further amplify users’ sense of vulnerability regarding personal information because personalization depends on the continuous collection and interpretation of user data. Moreover, social media platforms pose distinct privacy risks because disclosed personal information can be misused for malicious purposes, including phishing attacks and identity deception (Xie & Karan, 2019). These risks may become more salient when algorithmic personalization relies on extensive and ongoing data collection (Shin et al., 2022). Accordingly, we propose the following hypothesis.

**Hypothesis** **1.** *Perceived time spent on TikTok is positively associated with users’ privacy concerns.*

#### 2.1.2. Perceived Time Spent on TikTok and Health Consciousness

Health consciousness generally refers to individuals’ attentiveness to health-related issues and their motivation to maintain and protect their well-being (An et al., 2025; Cao et al., 2024). In many studies, health consciousness is treated as a relatively stable individual tendency. In the present study, however, we focus on health consciousness in the context of TikTok use. Specifically, we conceptualize it as a health-related awareness response that may become more salient when users perceive that they spend substantial time on the platform.

Perceived time spent on TikTok may increase users’ awareness of the potential health-related costs of platform use. Prolonged screen time has been associated with sedentary behavior, postural problems, and eye strain (Ellis Sandoval et al., 2025). Excessive social media use has also been linked to adverse psychological experiences, including anxiety, depression, and diminished self-esteem related to social comparison (Schmuck et al., 2019). Users who perceive themselves as spending more time on TikTok may therefore report greater attentiveness to possible health-related issues associated with platform use, including physical condition and psychological well-being.

This relationship may be particularly relevant in the TikTok context. TikTok’s continuous scrolling interface and algorithmically curated recommendations can facilitate prolonged and sometimes unintended use (Jiang et al., 2025). As users become more aware of the time they spend on the platform, they may also become more conscious of its potential implications for their health and well-being. Accordingly, we propose the following hypothesis.

**Hypothesis** **2.** *Perceived time spent on TikTok is positively associated with users’ health-related concerns.*

#### 2.1.3. Perceived Time Spent on TikTok and Social Interaction Anxiety

Social interaction anxiety refers to discomfort, anxiety, or fear that individuals may experience in social interactions, particularly when they feel exposed to interpersonal evaluation (Coelho & Romão, 2018; X. Zhang et al., 2019). On TikTok, social interaction is embedded in the platform’s design. Users are regularly exposed to visible engagement cues, such as likes, comments, and shares, which can make social evaluation and interpersonal visibility more salient (Cheng & Li, 2024). In social media contexts, pressure to maintain a favorable self-presentation and concerns about negative evaluation have been identified as important sources of psychological burden (Ou et al., 2023). The public nature of TikTok interactions, where comments and reactions can be visible to a broad audience, may further heighten users’ sensitivity to social evaluation (Medranda-Morales & Sakihama-Miyashiro, 2026).

Perceived time spent on TikTok may increase social interaction anxiety by making users more aware of these social-evaluative costs. Users who perceive that they spend more time on TikTok may feel more frequently exposed to engagement metrics, social comparison cues, and interactional expectations. These experiences can make interpersonal evaluation more salient and may contribute to anxiety in social interaction contexts. On this basis, the present study proposes the following hypothesis.

**Hypothesis** **3.** *Perceived time spent on TikTok is positively associated with users’ social interaction anxiety.*

#### 2.1.4. Perceived Time Spent on TikTok and Social Media Fatigue

Social media fatigue refers to users’ feelings of exhaustion, tiredness, or reduced motivation to continue using social media when platform use becomes overwhelming or excessive (Bright et al., 2015). Prior research suggests that social media fatigue can arise from information overload, emotional exhaustion, and the cognitive burden of managing social interactions, often referred to as social overload (Bright et al., 2015; Ou et al., 2023).

In the TikTok context, algorithmically curated recommendations, continuous scrolling, and autoplay functions can facilitate prolonged content consumption by reducing natural stopping points (Y. Xu et al., 2025). These platform characteristics may expose users to a continuous stream of content and interaction cues, which can make the cognitive and emotional costs of platform use more salient. Users who perceive that they spend more time on TikTok may therefore become more aware of the attentional and emotional effort involved in sustained platform engagement. This perceived effort may contribute to social media fatigue. Therefore, we propose the following hypothesis.

**Hypothesis** **4.** *Perceived time spent on TikTok is positively associated with users’ social media fatigue.*

### 2.2. Perceived Time Spent on TikTok and Overall User Satisfaction

Beyond its associations with psychological cost-related responses, perceived time spent on TikTok may also be positively associated with overall user satisfaction. Prior research suggests that users’ evaluations of digital platforms are shaped not only by potential costs but also by the extent to which platform use fulfills personal needs and provides emotional gratification (W. Zhang et al., 2023). From the perspective of Uses and Gratifications Theory, users actively engage with media platforms to satisfy needs such as entertainment, information seeking, and social sharing (Kaur et al., 2020; Kim et al., 2021). Users who perceive themselves as spending more time on TikTok may do so because the platform provides content experiences that they find enjoyable, personally relevant, or socially meaningful, which may be reflected in more favorable overall evaluations.

TikTok’s algorithmically driven content delivery system provides highly personalized content that aligns with users’ preferences and interaction patterns (Kang & Lou, 2022). As users perceive themselves as spending more time on the platform, this perception may reflect a stronger sense of immersion, enjoyment, and gratification derived from the content experience. Research on video-on-demand services has similarly shown that enjoyment is an important predictor of continued platform use and satisfaction (Pereira & Tam, 2021). In the TikTok context, where content is personalized and continuously refreshed, perceived time spent on TikTok may therefore indicate satisfaction-enhancing engagement with the platform.

In addition, perceived time spent may reflect users’ familiarity with TikTok and their habitual engagement with its content environment. As users repeatedly engage with the platform, they may become more attuned to the types of content that match their preferences, which can reinforce positive evaluations of the platform. Accordingly, we propose the following hypothesis.

**Hypothesis** **5.** *Perceived time spent on TikTok is positively associated with overall user satisfaction.*

### 2.3. Psychological Cost-Related Responses and Overall User Satisfaction

Privacy concerns may be negatively associated with overall user satisfaction because they can weaken users’ trust in the platform. When users feel that their personal data may be collected, monitored, or misused, their evaluation of the platform experience may become less favorable (Gelibolu & Mouloudj, 2025; Singh et al., 2024). In the TikTok context, algorithmic personalization depends on the continuous collection and interpretation of user data (Jiang et al., 2025). Thus, heightened privacy concerns may make privacy-related costs more salient and reduce users’ overall satisfaction with the platform.

Health consciousness may also be negatively associated with overall user satisfaction when users become more attentive to the potential health-related costs of platform use. In the context of TikTok, health consciousness reflects users’ awareness of possible implications for physical condition and psychological well-being. When users become more conscious of these potential costs, an otherwise enjoyable platform experience may be accompanied by concern about prolonged screen-based use. Because prolonged screen time has been associated with sedentary behavior, postural problems, and eye strain (Ellis Sandoval et al., 2025), heightened health consciousness may be associated with less favorable platform evaluations.

Social interaction anxiety may be negatively associated with overall user satisfaction because it can make platform interactions feel psychologically demanding. Users who experience anxiety about negative evaluation, social performance, or public visibility may perceive TikTok interactions as less socially rewarding (Guo & Au, 2026; Wu et al., 2025). Because TikTok includes visible interaction cues such as likes, comments, and shares, anxiety surrounding social interaction may reduce users’ overall satisfaction with the platform.

**Figure 1:**
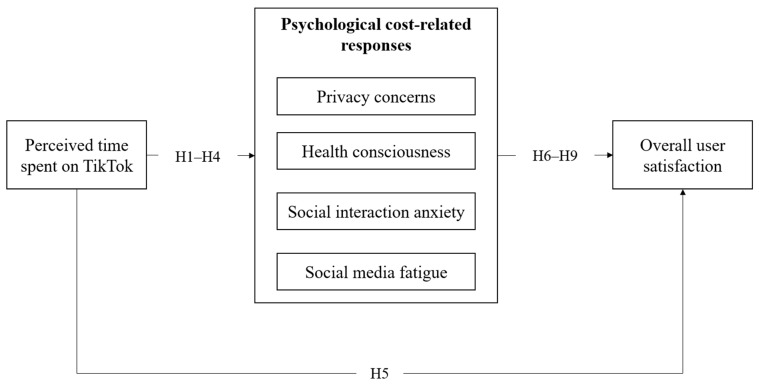
Conceptual framework of this research. Source: author’s drawing.

Social media fatigue may also be negatively associated with overall user satisfaction because it reflects exhaustion, tiredness, or reduced motivation to continue engaging with social media. As fatigue increases, TikTok may be perceived less as a source of entertainment and connection and more as a source of cognitive or emotional burden (Ou et al., 2023). Prior research has shown that social media fatigue is negatively associated with subjective well-being and continuance intention (Feng et al., 2024; Zhu & Yao, 2025). In the TikTok context, where continuous scrolling and algorithmically curated recommendations can facilitate prolonged content consumption, fatigue-related costs may be associated with lower overall user satisfaction. Therefore, we propose the following hypotheses and conceptual framework, as shown in Figure 1.

**Hypothesis** **6.** *Users’ privacy concerns are negatively associated with overall user satisfaction.*

**Hypothesis** **7.** *Users’ health consciousness is negatively associated with overall user satisfaction.*

**Hypothesis** **8.** *Users’ social interaction anxiety is negatively associated with overall user satisfaction.*

**Hypothesis** **9.** *Users’ social media fatigue is negatively associated with overall user satisfaction.*

## 3. Research Methodology

### 3.1. Measurement Development and Data Analysis

To enhance contextual validity, all items were adapted to reflect the TikTok usage environment. This adaptation was intended to align the measurement content with participants’ experiences on TikTok rather than with social media use in general. Perceived time spent on TikTok was measured using items adapted from Ernala et al. (2022). These items included seven-point Likert-type items and one 0–100 slider item assessing participant’s perceived amount of usual TikTok use. To capture psychological cost-related responses, we measured four distinct constructs: privacy concerns, health consciousness, social interaction anxiety, and social media fatigue. The corresponding items were adapted from Dinev and Hart (2005), Teng and Lu (2016), Alkis et al. (2017) and X. Zhang et al. (2019), and Bright et al. (2022), respectively. Finally, overall user satisfaction was measured using items adapted from Sharabati et al. (2022). Except for the 0–100 slider item used to measure perceived time spent on TikTok, all items were measured using seven-point Likert-type response scales, as shown in Appendix A.1.

Internal consistency was assessed using Cronbach’s alpha in SPSS 31.0. Confirmatory factor analysis (CFA) was then conducted to evaluate convergent validity, discriminant validity, and overall model fit. To evaluate the potential for common method bias, Harman’s single-factor test was performed, and variance inflation factors (VIFs) were examined as an additional diagnostic check. Hypotheses were tested using structural equation modeling (SEM) in AMOS 26.0. Complementing the SEM findings, fuzzy-set qualitative comparative analysis (fsQCA) was employed to identify configurational pathways associated with high overall user satisfaction. This analysis offered a set-theoretic perspective on how combinations of perceived time spent on TikTok and psychological cost-related responses were associated with high overall user satisfaction.

### 3.2. Data Collection and Sample Characteristics

We administered an online survey and recruited respondents through Prolific. Before the main analysis, we applied data-screening procedures to enhance response quality. Prior to the survey, participants completed a screening question asking how long they had been using TikTok, with response options ranging from “Never” to “More than 24 months.” Respondents who selected “Never” were screened out before completing the survey, so the final sample included participants with prior TikTok experience. A total of 326 responses were initially collected. Cases with completion times of less than 1 min were removed (*n* = 20), leaving 306 usable responses for analysis.

All respondents were U.S. residents aged 18 years or older. As reported in Table 1, the sample included respondents with varied demographic characteristics. Specifically, 50.3% of respondents identified as female, and the mean age was 43.64 years. Regarding TikTok usage characteristics, 50.3% of participants reported using TikTok for more than 24 months, and the largest proportion identified themselves as viewers/likers (85.6%).

## 4. Results

### 4.1. Measurement Assessment

Before conducting CFA, we performed an exploratory factor analysis to examine the structure of the measurement items. Principal component extraction with Varimax rotation was used as an initial diagnostic procedure. The results indicated that the data were suitable for factor analysis. Specifically, the Kaiser–Meyer–Olkin measure of sampling adequacy was high (KMO = 0.885), exceeding the recommended cutoff, and Bartlett’s test of sphericity was significant (χ^2^ = 9022.285, df = 406, *p* < 0.001), supporting the suitability of the data for factor analysis, as shown in Appendix A.2.

We then performed CFA to assess the measurement model. One item, HC5, was excluded because its standardized factor loading was below the recommended cutoff of 0.50. The final measurement model included six multi-item constructs: perceived time spent on TikTok, privacy concerns, health consciousness, social interaction anxiety, social media fatigue, and overall user satisfaction. Perceived time spent on TikTok was measured with 4 items, privacy concerns with 4 items, health consciousness with 5 items, social interaction anxiety with 6 items, social media fatigue with 4 items, and overall user satisfaction with 5 items. Cronbach’s alpha was computed for each construct, as shown in Table 2.

The measurement model demonstrated acceptable fit: χ^2^ = 1008.566, df = 335, χ^2^/df = 3.011, IFI = 0.923, TLI = 0.906, CFI = 0.922, and RMSEA = 0.075. All retained items had standardized factor loadings greater than 0.50, supporting convergent validity. Convergent validity and construct reliability were further supported because all average variance extracted values exceeded 0.50 and all composite reliability values were above 0.70 (Fornell & Larcker, 1981). Internal consistency was also satisfactory, as Cronbach’s alpha values exceeded 0.80 for all constructs, indicating strong reliability (Al-Abdallah et al., 2026).

Finally, discriminant validity was assessed using the Fornell–Larcker criterion. As shown in Table 3, the square root of each construct’s AVE was greater than its correlations with the other constructs, supporting discriminant validity.

### 4.2. Common Method Bias

For the analyzed sample (*n* = 306), Harman’s single-factor test showed that the first factor explained 29.83% of the total variance, which was below the commonly used threshold of 50%. Because Harman’s single-factor test provides only a limited diagnostic assessment of common method bias, we also examined variance inflation factors (VIFs) for the study constructs. All VIF values were below 3, ranging from 1.165 to 1.724. These results do not eliminate the possibility of common method bias, but they suggest that it was unlikely to be a dominant source of bias in the present analysis (Podsakoff et al., 2003; Zhou & Liu, 2026).

### 4.3. Structural Model Assessment

SEM was conducted using AMOS 26.0 to examine the proposed associations. The final structural model showed a marginally acceptable fit to the data: χ^2^ = 1181.977, df = 341, CMIN/DF = 3.466, *p* < 0.001, IFI = 0.904, TLI = 0.893, CFI = 0.903, and RMSEA = 0.090. Although the chi-square statistic was significant, which is common in complex models with moderately large samples, both CFI and IFI exceeded the recommended threshold of 0.90. TLI was slightly below 0.90, and RMSEA exceeded the conventional cutoff of 0.08. Taken together, these results indicate that the structural model fit was marginal but acceptable for examining the hypothesized associations.

The results of the structural model analysis are presented in Table 4. Perceived time spent on TikTok was not significantly associated with privacy concerns (β = 0.038, *p* = 0.641), indicating that H1 was not supported. Perceived time spent on TikTok was significantly and positively associated with health consciousness (β = 0.399, *p* < 0.001), supporting H2. Perceived time spent on TikTok was also significantly and positively associated with social interaction anxiety (β = 0.281, *p* < 0.001), supporting H3. However, perceived time spent on TikTok was not significantly associated with social media fatigue (β = 0.073, *p* = 0.240), indicating that H4 was not supported. Furthermore, perceived time spent on TikTok was significantly and positively associated with overall user satisfaction (β = 0.530, *p* < 0.001), supporting H5. This result suggests that perceived time spent on TikTok was positively associated with overall user satisfaction, even after accounting for the psychological cost-related responses included in the model.

**Figure 2:**
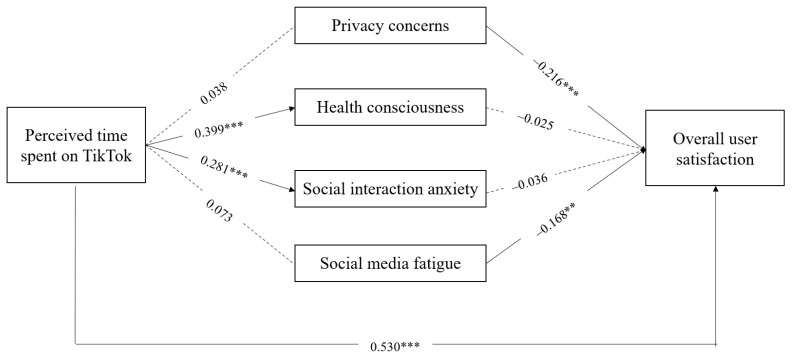
SEM result.

Regarding the associations between psychological cost-related responses and overall user satisfaction, privacy concerns were significantly and negatively associated with overall user satisfaction (β = −0.216, *p* < 0.001), supporting H6. Health consciousness was not significantly associated with overall user satisfaction (β = −0.025, *p* = 0.659), indicating that H7 was not supported. Social interaction anxiety was also not significantly associated with overall user satisfaction (β = −0.036, *p* = 0.502), indicating that H8 was not supported. Social media fatigue was significantly and negatively associated with overall user satisfaction (β = −0.168, *p* = 0.002), supporting H9. Overall, the structural model results provide partial support for the proposed hypotheses, as shown in Figure 2.

### 4.4. Fuzzy-Set Qualitative Comparative Analysis (fsQCA)

We applied fuzzy-set qualitative comparative analysis (fsQCA) to identify configurations of conditions associated with both the presence and absence of overall user satisfaction. Grounded in Boolean set theory and algebra, fsQCA integrates qualitative and quantitative reasoning through a configurational perspective that accommodates complexity and equifinality. This approach recognizes that the same outcome may be associated with multiple and substantively different pathways (Ragin, 2009). Unlike variable-centered techniques, which emphasize independent net effects, fsQCA conceptualizes outcomes as arising from interdependent conditions that operate jointly.

**Figure 3:**
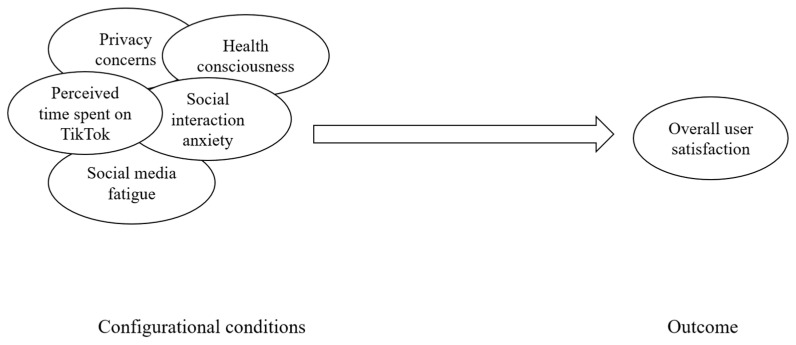
fsQCA research framework.

From an analytical standpoint, fsQCA differentiates between necessary and sufficient conditions. Necessary conditions are conditions that must be present for an outcome to occur, although they are not sufficient by themselves. Sufficient conditions are conditions that, either alone or in combination with other conditions, are consistently associated with the outcome (Ragin, 2009). Because overall user satisfaction may be linked to multiple alternative configurations, fsQCA is useful for identifying empirically supported combinations of conditions associated with the focal outcome. Accordingly, we used fsQCA to examine how perceived time spent on TikTok and four psychological cost-related responses, namely privacy concerns, health consciousness, social interaction anxiety, and social media fatigue, were jointly associated with overall user satisfaction, as shown in Figure 3.

#### 4.4.1. Calibration

All condition variables and the outcome variable were calibrated using the direct method. For each variable, three anchor points were specified. The 95th percentile was used as the threshold for full membership, the 50th percentile as the crossover point, and the 5th percentile as the threshold for full non-membership. These percentile-based anchors were used to transform the Likert-type scale data into fuzzy-set membership scores, following prior fsQCA applications using survey-based measures (Yu et al., 2025). The specific anchor values for each condition and the outcome are reported in Table 5.

#### 4.4.2. Necessary Conditions Analysis

We conducted a necessary conditions analysis to evaluate whether any single condition consistently accompanied overall user satisfaction. In fsQCA, consistency captures how reliably a condition is present when the outcome occurs, whereas coverage indicates the empirical relevance of the condition by showing the proportion of outcome cases associated with it. Following established guidelines, a condition is typically treated as necessary only when its consistency is at least 0.90 and its coverage exceeds 0.75 (Elshaer et al., 2024). The results indicate that none of the examined conditions reached the 0.90 consistency threshold. Accordingly, no single condition can be considered necessary for overall user satisfaction, as shown in Table 6.

#### 4.4.3. Sufficient Conditions Analysis

To identify combinations of conditions associated with high overall user satisfaction, we constructed truth tables using fsQCA software. With five condition variables, the analysis yielded 32 logically possible configurations. Each configuration was evaluated in terms of frequency, consistency, and coverage. Frequency reflects the number of cases represented by a configuration, consistency indicates the degree to which a configuration is consistently associated with the outcome, and coverage captures the empirical relevance of the configuration for the outcome (Ragin, 2009). The frequency threshold was set to 1, and the consistency cutoff was set to 0.85 (Prado-Gascó et al., 2017).

Among the three solution types provided by fsQCA, namely complex, parsimonious, and intermediate solutions, we report the intermediate solution because it offers a practical balance between theoretical meaningfulness and interpretability (Shui et al., 2025). Table 7 summarizes the resulting configurations. In the solution table, a filled circle (●) denotes the presence of a condition, a crossed circle (⊗) denotes the absence of a condition, and blank cells indicate that the condition is not specified in that configuration.

The analysis produced six distinct configurational pathways associated with high overall user satisfaction, which is consistent with the principle of equifinality. The configuration-level consistency values ranged from 0.883 to 0.924. The overall solution consistency was 0.864, and the overall solution coverage was 0.628. These results indicate that high overall user satisfaction was associated with multiple configurations of perceived time spent on TikTok and psychological cost-related responses.

Solution 1 is characterized by the presence of high perceived time spent on TikTok and the absence of health consciousness and social media fatigue. This configuration suggests that high satisfaction can occur when users perceive substantial time spent on TikTok but do not report heightened health-related awareness or fatigue-related costs.

Solution 2 is characterized by the presence of high perceived time spent on TikTok and the absence of privacy concerns and social interaction anxiety. This configuration suggests that high satisfaction can occur when perceived time spent is high while privacy-related and social-evaluative costs are low.

Solution 3 involves the presence of high perceived time spent on TikTok and health consciousness, combined with the absence of privacy concerns. This configuration suggests that high satisfaction can coexist with health-related awareness when privacy concerns are absent.

Solution 4 is characterized by the presence of high perceived time spent on TikTok and social interaction anxiety, combined with the absence of social media fatigue. This configuration suggests that high satisfaction can coexist with social interaction anxiety when fatigue-related costs are absent.

Solution 5 involves the presence of high perceived time spent on TikTok, health consciousness, and social media fatigue, combined with the absence of social interaction anxiety. This configuration suggests that high satisfaction can occur even when health-related awareness and fatigue are present, provided that social interaction anxiety is absent.

Solution 6 is characterized by the absence of high perceived time spent on TikTok, privacy concerns, and health consciousness, combined with the presence of social interaction anxiety and social media fatigue. This configuration suggests that high satisfaction can occur under a lower perceived-time-spent condition when privacy-related and health-related cost responses are absent, even if social interaction anxiety and fatigue are present.

Notably, high perceived time spent on TikTok appears as a presence condition in Solutions 1, 2, 3, 4, and 5. This pattern is consistent with the SEM finding that perceived time spent on TikTok is positively associated with overall user satisfaction. The absence of social media fatigue appears in Solutions 1 and 4, which is also consistent with the SEM finding that social media fatigue is negatively associated with overall user satisfaction. Collectively, these six configurations provide a configurational account of high overall user satisfaction by showing that it can be associated with multiple combinations of perceived time spent and psychological cost-related responses rather than a single dominant condition.

To complement the high-satisfaction analysis, we also examined configurations associated with low overall user satisfaction, as shown in Table 8. The analysis identified two configurations, with an overall consistency of 0.884 and an overall coverage of 0.400.

Solution 1 is characterized by the absence of high perceived time spent on TikTok, health consciousness, and social interaction anxiety, combined with the presence of privacy concerns and social media fatigue. This configuration suggests that low overall user satisfaction can occur when users report privacy-related concerns and fatigue-related costs, even when perceived time spent, health-related awareness, and social interaction anxiety are low.

Solution 2 involves the presence of high perceived time spent on TikTok, social interaction anxiety, and social media fatigue, combined with the absence of privacy concerns and health consciousness. This configuration suggests that low overall user satisfaction can occur even under high perceived time spent when social-evaluative and fatigue-related costs are present.

Notably, social media fatigue appears as a presence condition in both configurations. This pattern is broadly consistent with the SEM finding that social media fatigue is negatively associated with overall user satisfaction. Collectively, these configurations suggest that low overall user satisfaction is consistently linked to fatigue-related psychological costs across different patterns of perceived TikTok use. These findings also highlight the asymmetric nature of the conditions associated with high and low overall user satisfaction.

## 5. Discussion

This study examined how perceived time spent on TikTok is associated with overall user satisfaction and psychological cost-related responses. The results provide partial support for the proposed relationships and suggest that perceived time spent on TikTok has a dual pattern of associations. On the one hand, it is positively associated with overall user satisfaction. On the other hand, it is selectively associated with specific psychological cost-related responses, particularly health consciousness and social interaction anxiety.

Although the structural model showed marginal fit, the SEM results provide useful information about the proposed associations. Specifically, perceived time spent on TikTok is significantly and positively associated with health consciousness and social interaction anxiety, whereas its associations with privacy concerns and social media fatigue are not statistically significant. This pattern suggests that greater perceived time spent on TikTok is more closely related to users’ awareness of health-related issues and social-evaluative concerns than to privacy-related concerns or fatigue-related costs. One possible explanation is that short-video consumption is characterized by brief, fragmented, and rapidly shifting interactions (Jiang et al., 2025). These interactions may be linked to users’ awareness of their own media consumption and social visibility, but they may not necessarily correspond to heightened privacy concerns or fatigue for all users. Thus, perceived time spent does not appear to be uniformly associated with all psychological cost-related responses.

Perceived time spent on TikTok is also directly and positively associated with overall user satisfaction. This finding suggests that users’ subjective sense of spending time on TikTok may reflect satisfaction-enhancing engagement with the platform. From the perspective of Uses and Gratifications Theory, users actively engage with media platforms to satisfy needs such as entertainment, information seeking, and social sharing (Kaur et al., 2020; Kim et al., 2021). Users who perceive themselves as spending more time on TikTok may therefore experience greater gratification from personalized, entertaining, and socially relevant content, which may be reflected in higher overall user satisfaction.

The associations between psychological cost-related responses and overall user satisfaction are also heterogeneous. Privacy concerns are significantly and negatively associated with overall user satisfaction, indicating that perceived risks related to personal data remain important in users’ overall evaluations of TikTok. Social media fatigue is also significantly and negatively associated with overall user satisfaction, suggesting that exhaustion and reduced motivation to continue using the platform are associated with less favorable evaluations. These findings indicate that privacy-related and fatigue-related costs are evaluatively relevant psychological costs in the TikTok context.

In contrast, health consciousness is not significantly associated with overall user satisfaction. This finding suggests that health consciousness is not significantly associated with users’ overall evaluations of TikTok, even though it is positively associated with perceived time spent. One possible explanation is that health consciousness has a dual character in this context. It may reflect awareness of potential health-related costs, but it may also indicate a more adaptive form of self-monitoring or self-regulation. This dual role may distinguish health consciousness from more clearly negative cost-related responses, such as privacy concerns and social media fatigue. Social interaction anxiety is also not significantly associated with overall user satisfaction. Although perceived time spent is associated with higher social interaction anxiety, this anxiety does not necessarily translate into lower platform satisfaction. TikTok’s short-video format and passive consumption options, such as scrolling and watching without posting or commenting, may allow users to experience social-evaluative concerns without allowing those concerns to dominate their overall evaluation of the platform.

The fsQCA results complement the SEM findings by revealing multiple configurations associated with high overall user satisfaction. This pattern is consistent with the principle of equifinality. High perceived time spent on TikTok appears as a presence condition in Solutions 1, 2, 3, 4, and 5, which is consistent with the SEM finding that perceived time spent is positively associated with overall user satisfaction. The absence of privacy concerns appears in multiple configurations, which is also consistent with the SEM finding that privacy concerns are negatively associated with overall user satisfaction. Similarly, the absence of social media fatigue appears in Solutions 1 and 4, which aligns with the significant negative association between social media fatigue and overall user satisfaction. In contrast, health consciousness and social interaction anxiety appear across configurations in both their presence and absence, reflecting their more conditional roles. This pattern is consistent with their non-significant associations with overall user satisfaction in the SEM analysis. Collectively, the fsQCA results show that high overall user satisfaction is not linked to a single dominant condition but is associated with multiple combinations of perceived time spent on TikTok and psychological cost-related responses.

The SEM and fsQCA findings are analytically complementary. The SEM results identify average population-level associations, showing that perceived time spent on TikTok is positively associated with overall user satisfaction, whereas privacy concerns and social media fatigue are negatively associated with overall user satisfaction. The fsQCA results extend this interpretation by showing that high overall user satisfaction can occur through multiple configurations rather than through one uniform pattern. In particular, the repeated presence of high perceived time spent across five of the six high-satisfaction configurations reinforces the role of perceived time spent as a key condition associated with favorable platform evaluations. At the same time, the configurational results show that psychological cost-related responses do not operate in a uniform manner. Some configurations include the absence of specific costs, whereas others show that high satisfaction can coexist with certain cost-related responses.

Taken together, these findings suggest that psychological responses to TikTok use are multifaceted and vary in their associations with overall user satisfaction. Some psychological cost-related responses, particularly privacy concerns and social media fatigue, are negatively associated with users’ evaluations of the platform. Others, such as health consciousness and social interaction anxiety, appear to reflect psychological responses that may accompany perceived time spent but are not necessarily negatively associated with satisfaction. This pattern suggests that perceived time spent on TikTok may be positively associated with overall user satisfaction while also coexisting with specific psychological costs. By combining SEM and fsQCA, the present study provides a more nuanced account of how perceived time spent on TikTok and psychological cost-related responses are associated with overall user satisfaction in short-video platform use.

### 5.1. Theoretical Contribution

First, this study extends research on short-video platform use by examining perceived time spent on TikTok in relation to both overall user satisfaction and psychological cost-related responses. Prior research on short-video and social media platforms has examined user engagement (Xiao et al., 2026; Z. Zhang et al., 2025), hedonic motivation and continuance intention (Deng & Yu, 2023; Yang et al., 2023), and problematic TikTok use (Jain et al., 2025). However, less is known about how users’ subjective perception of time spent is associated with both overall user satisfaction and psychological cost-related responses. By conceptualizing perceived time spent as a psychologically meaningful indicator of subjective platform engagement, this study shows that perceived time spent can be positively associated with satisfaction while also being selectively associated with privacy-, health-, social-, and fatigue-related costs.

Second, this study advances the conceptualization of perceived time spent on TikTok as a psychologically meaningful construct that differs from objective usage metrics. Existing research on social media use has often relied on actual screen time or self-reported frequency as indicators of engagement (Yoon et al., 2019), treating usage duration primarily as a descriptive or background variable. By focusing on users’ subjective perception of their own time spent, this study treats perceived time spent as a meaningful indicator of users’ perceived intensity of TikTok use. Prior research on time perception suggests that individuals may not accurately recall the duration of engaging activities (Jiang et al., 2025; Tobin & Grondin, 2009). Building on this insight, the present study suggests that perceived time spent may be closely linked to users’ psychological responses and evaluative judgments in short-video consumption contexts. This perspective opens a useful avenue for integrating subjective time perception into social media research and suggests that perceived usage awareness may be an important factor in understanding users’ platform evaluations.

Third, this study contributes to the literature by demonstrating the heterogeneous and selective nature of psychological cost-related responses to perceived TikTok use. Rather than assuming that perceived time spent is uniformly associated with all forms of psychological cost, the findings show that it is significantly associated with health consciousness and social interaction anxiety, but not with privacy concerns or social media fatigue. Likewise, the associations between psychological cost-related responses and overall user satisfaction are selective. Privacy concerns and social media fatigue are negatively associated with overall user satisfaction, whereas health consciousness and social interaction anxiety are not. These findings suggest that psychological costs in short-video platform use should not be treated as a single uniform construct. Instead, privacy concerns, health consciousness, social interaction anxiety, and social media fatigue capture distinct psychological responses with different evaluative implications. This differentiated pattern refines our understanding of social media use by showing that perceived time spent may be linked to some psychological cost-related responses but not others, and that not all cost-related responses necessarily reduce overall user satisfaction.

Finally, this study contributes by showing that perceived time spent on TikTok is not only associated with psychological cost-related responses but also directly and positively associated with overall user satisfaction. The significant positive association suggests that perceived time spent may reflect engagement, gratification, or perceived value derived from platform use. This finding qualifies a purely cost-based interpretation of TikTok use and points to the need for a more balanced theoretical account that considers both the gratification-related benefits and psychological costs of perceived platform engagement. Perceived time spent on TikTok may therefore show dual associations: a positive association with overall user satisfaction and coexistence with specific psychological cost-related responses. This perspective offers a more nuanced account of perceived TikTok use in algorithm-driven short-video environments.

### 5.2. Managerial Implications

This study provides several managerial insights for social media platforms, particularly short-video platforms such as TikTok. First, the findings show that perceived time spent on TikTok is positively associated with overall user satisfaction. This association suggests that users’ experience of time spent on the platform is relevant to their overall evaluation, but it should not be interpreted as a recommendation to maximize usage duration. Platform managers should therefore focus on improving the quality, relevance, and transparency of content experiences while preserving users’ sense of control. Features that support meaningful content discovery, personalized recommendations, and enjoyable browsing may help sustain satisfaction without encouraging excessive use.

Second, the findings highlight the importance of privacy management in maintaining overall user satisfaction. Although perceived time spent on TikTok is not significantly associated with privacy concerns, privacy concerns are significantly and negatively associated with overall user satisfaction. This suggests that users remain sensitive to potential risks associated with personal data collection and algorithmic tracking. Platform managers should therefore enhance transparency in data usage policies, provide clearer explanations of recommendation algorithms, and offer users greater control over privacy settings. Such practices may help build trust and reduce the negative association between privacy concerns and the user experience.

Third, the findings suggest that platforms should carefully manage fatigue-related psychological costs. Social media fatigue is negatively associated with overall user satisfaction, indicating that exhaustion, tiredness, and reduced motivation are associated with less favorable evaluations of the platform. Short-video platforms should therefore consider design features that help users regulate prolonged engagement, such as optional stopping cues, session reminders, content variety controls, or digital well-being dashboards. These features may help users manage their engagement while preserving the entertainment value of the platform.

Fourth, the findings indicate that platforms may benefit from supporting mindful and responsible usage behaviors. Although health consciousness is not significantly associated with overall user satisfaction, perceived time spent on TikTok is positively associated with health consciousness. This suggests that users who perceive themselves as spending more time on TikTok may become more aware of the possible physical and psychological implications of platform use. Features such as screen-time summaries, activity dashboards, and voluntary usage reminders may help users reflect on their media consumption. Such tools can support users’ sense of control without necessarily undermining their satisfaction with the platform.

Fifth, the findings suggest that low-pressure interaction environments may help maintain favorable user evaluations. Social interaction anxiety is not significantly associated with overall user satisfaction, suggesting that TikTok’s passive and entertainment-oriented consumption mode may allow users to experience the platform without requiring intensive social interaction or self-presentation. Platform designers should therefore continue to support flexible engagement patterns that allow users to watch, browse, and interact at different levels of visibility and participation. This approach may help maintain a comfortable and accessible user experience.

### 5.3. Limitations and Future Research

Despite its contributions, this study has several limitations that open avenues for future research. First, this study relied on self-reported survey data, which may involve common method variance and perceptual inaccuracy. Although Harman’s single-factor test and variance inflation factors suggested that common method variance was unlikely to be a severe concern, all variables were collected from the same respondents at a single point in time. Future research should employ procedural remedies, such as temporal separation between predictor and outcome measurement, or incorporate objective behavioral data to reduce this concern. In particular, perceived time spent on TikTok captures users’ subjective assessment of their platform use rather than objectively observed behavior. Future research could incorporate objective usage indicators, such as digital trace data or actual screen-time records, to provide a more precise and triangulated understanding of how actual usage patterns and perceived time spent are associated with psychological cost-related responses and platform evaluations.

Second, the model specification should be interpreted with caution. The structural model demonstrated marginal fit, with RMSEA exceeding the conventional cutoff of 0.08. Future research should further refine the model by incorporating additional constructs that may better explain how perceived time spent on TikTok is associated with both overall user satisfaction and psychological cost-related responses. Potential constructs include perceived enjoyment, gratification fulfillment, algorithmic trust, perceived loss of control, fear of missing out, and perceived autonomy.

Third, several psychological cost-related responses were not significantly associated with overall user satisfaction, and these non-significant findings warrant further examination. Health consciousness may not function as a purely negative psychological cost because it may also reflect adaptive self-monitoring or responsible usage awareness. Similarly, social interaction anxiety may not necessarily reduce overall satisfaction because TikTok allows passive consumption, such as scrolling and watching videos without posting, commenting, or directly interacting with others. Future research should examine the boundary conditions under which health consciousness and social interaction anxiety become evaluatively consequential, including usage motives, content type, posting behavior, and the degree of public visibility in platform interactions.

Fourth, although this study examined four psychological cost-related responses, other relevant psychological responses may also be associated with users’ evaluations of short-video platforms. For example, perceived enjoyment, algorithmic trust, fear of missing out, perceived content relevance, self-control concerns, attentional depletion, addictive tendencies, and perceived autonomy may be associated with how users evaluate their TikTok experience. Incorporating these constructs would help develop a more comprehensive account of the positive and negative psychological responses of short-video platform use.

Fifth, this study has methodological and measurement limitations. EFA and CFA were conducted using the same sample, which may limit the stability of the measurement validation. Future research should validate the measurement model using independent samples or employ a split-sample approach. In addition, perceived time spent on TikTok was measured as a broad subjective construct that captures related aspects of perceived use, including perceived amount of use, frequency, and comparison with others. Although this approach was useful for capturing users’ overall subjective sense of TikTok use, future research should develop and validate more rigorous measures that distinguish perceived duration, perceived frequency, perceived intensity, and comparative usage perceptions in short-video platform contexts. Some TikTok-specific item adaptations should also be interpreted with caution. Some TikTok-specific item adaptations should also be interpreted with caution. The social interaction anxiety items were originally developed to capture anxiety in broader interpersonal or offline-based social interaction contexts, and therefore may not fully reflect TikTok-specific forms of social discomfort, such as social comparison, visibility concerns, or evaluation anxiety that can occur without direct interaction. In addition, the social media fatigue items were adapted from broader social media contexts and may not fully capture fatigue arising from TikTok-specific usage patterns, such as passive viewing, rapid scrolling, algorithmic exposure, and continuous short-form content consumption. Future research should develop and validate short-video-platform-specific measures of psychological cost-related responses.

Sixth, the sample was confined to U.S. adults, which limits the cultural generalizability of the findings. Users from different cultural contexts may experience psychological cost-related responses differently, particularly privacy concerns and social interaction anxiety. Moreover, the screening question captured participants’ prior TikTok experience but did not directly verify whether they were regular or current TikTok users. Future research should use more precise screening criteria, such as current usage status, recent activity, or frequency of use, and conduct cross-cultural replication studies to examine whether the associations and configurations identified in this study generalize beyond U.S. adult users.

Seventh, the current sample consisted of general TikTok users whose usage may not constitute clinically meaningful overuse. Therefore, the findings should not be interpreted as evidence regarding problematic or addictive TikTok use. Future research should incorporate validated measures of problematic social media use or recruit high-risk user groups to examine whether the same pattern of associations emerges among users who experience more severe or clinically meaningful overuse.

Finally, as with all cross-sectional studies, the directional relationships assumed in the proposed model cannot be firmly established from the current data. Although this study conceptualized perceived time spent on TikTok as being associated with psychological cost-related responses and overall user satisfaction, alternative directional orderings remain plausible. For instance, individuals who are already more anxious, health-conscious, or privacy-sensitive may be more likely to notice or amplify their perception of time spent on TikTok. More broadly, psychological states may precede and shape patterns of social media engagement rather than simply follow from them. Future research should employ longitudinal panel designs, experimental designs, or experience sampling methods to establish stronger directional inference and disentangle potential bidirectional relationships among the constructs examined in this study.

## Figures and Tables

**Table 1:** Demographic and TikTok usage characteristics of the sample (*n* = 306).

Dimensions	Items	Frequency	Percentage (%)
Gender	Male	149	48.7
	Female	154	50.3
	Non-binary	2	0.7
	Prefer not to say	1	0.3
Age	18–24 years old	36	11.8
	25–35 years old	73	23.8
	36–45 years old	57	18.6
	46–55 years old	58	19
	Over 55 years old	82	26.8
Ethnicity	Caucasian	204	66.7
	African American	37	12.1
	Latino/Hispanic	35	11.4
	Asian	20	6.5
	Native Hawaiian or Pacific Islander	1	0.3
	Other	9	2.9
Annual Household Income	Less than $10,000	10	3.3
	$10,000–$19,999	13	4.2
	$20,000–$29,999	26	8.5
	$30,000–$39,999	24	7.8
	$40,000–$49,999	29	9.5
	$50,000–$59,999	30	9.8
	$60,000–$69,999	37	12.1
	$70,000–$79,999	24	7.8
	$80,000–$89,999	12	3.9
	$90,000–$99,999	19	6.2
	$100,000–$149,999	47	15.4
	More than $150,000	35	11.4
Education	Less than high school	1	0.3
	High school graduate	40	13.1
	Some college	84	27.5
	College degree	135	44.1
	Post graduate degree	46	15
TikTok Usage Period	1–6 months	39	12.7
	6–12 months	42	13.7
	12–24 months	71	23.2
	More than 24 months	154	50.3
Self-identified User Type	Viewer/Liker	262	85.6
	Creator/Content contributor	7	2.3
	Both	37	12.1

**Table 2:** Variables, estimate, average variance extracted (AVE), composite reliability (CR), and Cronbach’s alphas.

Construct Items		Estimate	AVE	CR	Cronbach’s Alpha
Perceived time spent on TikTok	Time1	0.757	0.756	0.925	0.892
	Time2	0.915			
	Time3	0.914			
	Time4	0.883			
Privacy Concerns	PC1	0.810	0.786	0.936	0.934
	PC2	0.786			
	PC3	0.970			
	PC4	0.963			
Health Consciousness	HC1	0.916	0.642	0.897	0.900
	HC2	0.917			
	HC3	0.865			
	HC4	0.677			
	HC6	0.569			
Social interaction anxiety	SIA1	0.929	0.867	0.975	0.975
	SIA2	0.953			
	SIA3	0.967			
	SIA4	0.943			
	SIA5	0.870			
	SIA6	0.923			
Social media fatigue	SMF1	0.818	0.637	0.874	0.871
	SMF2	0.881			
	SMF4	0.820			
	SMF5	0.657			
Overall user satisfaction	SA1	0.922	0.724	0.929	0.929
	SA2	0.841			
	SA3	0.800			
	SA4	0.883			
	SA5	0.803			

**Table 3:** Convergent and discriminant validity.

	Mean	SD	Time	PC	HC	SIA	SMF	SA
Time	2.819	0.904	0.869					
PC	4.238	1.606	0.014	0.886				
HC	3.235	1.490	0.328 **	0.168 **	0.801			
SIA	2.296	1.390	0.252 **	0.290 **	0.468 **	0.931		
SMF	3.389	1.449	0.024	0.356 **	0.271 **	0.429 **	0.789	
SA	4.890	1.156	0.401 **	−0.242 **	0.098	−0.006	−0.250 **	0.851

Notes: Time: perceived time spent on TikTok; PC: privacy concerns; HC: health consciousness; SIA: social interaction anxiety; SMF: social media fatigue; SA: overall user satisfaction; ** *p* < 0.01.

**Table 4:** The result of SEM (*n* = 306).

Hypotheses				B	S.E.	t-Value	Result
H1	Time	→	PC	0.038	0.085	0.641	Not supported
H2	Time	→	HC	0.399 ***	0.091	6.813	Supported
H3	Time	→	SIA	0.281 ***	0.084	4.813	Supported
H4	Time	→	SMF	0.073	0.090	1.176	Not supported
H5	Time	→	SA	0.530 ***	0.073	8.457	Supported
H6	PC	→	SA	−0.216 ***	0.043	−4.139	Supported
H7	HC	→	SA	−0.025	0.043	−0.442	Not supported
H8	SIA	→	SA	−0.036	0.043	−0.671	Not supported
H9	SMF	→	SA	−0.168 **	0.043	−3.133	Supported

Notes: Time: perceived time spent on TikTok; PC: privacy concerns; HC: health consciousness; SIA: social interaction anxiety; SMF: social media fatigue; SA: overall user satisfaction; *** *p* < 0.001; ** *p* < 0.01.

**Table 5:** Calibration using percentiles.

Calibration	Perceived Time Spent on TikTok	Privacy Concerns	Health Consciousness	Social Interaction Anxiety	Social Media Fatigue	Overall User Satisfaction
0.95	4.67	6.75	6.00	5.50	6.00	6.40
0.50	2.67	4.50	3.10	2.00	3.25	5.00
0.05	1.67	1.75	1.00	1.00	1.00	2.47

**Table 6:** Analysis of necessary conditions.

Condition	Consistency	Coverage
Time	0.694359	0.804709
~Time	0.596529	0.604201
PC	0.572102	0.643017
~PC	0.703428	0.732389
HC	0.650765	0.716911
~HC	0.632686	0.671329
SIA	0.562490	0.693840
~SIA	0.704033	0.677292
SMF	0.584679	0.630830
~SMF	0.707298	0.766027

Notes: ~: absence of condition; Time: perceived time spent on TikTok; PC: privacy concerns; HC: health consciousness; SIA: social interaction anxiety; SMF: social media fatigue.

**Table 7:** Analysis of sufficient conditions.

Configurational Conditions	Solution 1	Solution 2	Solution 3	Solution 4	Solution 5	Solution 6
Time	●	●	●	●	●	⊗
PC		⊗	⊗			⊗
HC	⊗		●		●	⊗
SIA		⊗		●	⊗	●
SMF	⊗			⊗	●	●
Consistency	0.918	0.914	0.908	0.922	0.924	0.883
Raw coverage	0.386	0.419	0.396	0.343	0.295	0.230
Unique coverage	0.026	0.013	0.032	0.020	0.021	0.031
Overall solution coverage	0.628					
Overall solution consistency	0.864					

Notes: ●: presence of condition; ⊗: absence of condition; Time: perceived time spent on TikTok; PC: privacy concerns; HC: health consciousness; SIA: social interaction anxiety; SMF: social media fatigue.

**Table 8:** Analysis of configurational conditions.

Configurational Conditions	Solution 1	Solution 2
Time	⊗	●
PC	●	⊗
HC	⊗	⊗
SIA	⊗	●
SMF	●	●
Consistency	0.929	0.866
Raw coverage	0.338	0.246
Unique coverage	0.155	0.062
Overall solution coverage	0.400	
Overall solution consistency	0.884	

Notes: ●: presence of condition; ⊗: absence of condition; Time: perceived time spent on TikTok; PC: privacy concern; HC: health consciousness; SIA: social interaction anxiety; SMF: social media fatigue.

## Data Availability

The data used in this study are available from the corresponding author upon reasonable requests.

## Appendix A

### Appendix A.1. Constructs and Measurement Items

**Constructs**	**Code**	**Items**	**References**
Perceived time spent on TikTok	Time1	How much time do you feel you spend on TikTok?	Ernala et al. (2022)
	Time2	How much do you usually use TikTok?	
	Time3	How much do you usually use TikTok? (continuous slider, 0–100)	
	Time4	How much do you think you use TikTok compared to other people?	
Privacy concerns (PC)	PC1	I am concerned that the information I submit on TikTok could be misused.	Dinev and Hart (2005)
	PC2	I am concerned that a person can find private information about me on TikTok.	
	PC3	I am concerned about submitting information on TikTok, because of what others might do with it.	
	PC4	I am concerned about submitting information on TikTok, because it could be used in a way I did not foresee.	
Health Consciousness (HC)	HC1	I reflect about my health a lot after using TikTok.	Teng and Lu (2016)
	HC2	I’m very self-conscious about my health after using TikTok.	
	HC3	I’m alert to changes in my health after using TikTok.	
	HC4	I’m usually aware of my health after using TikTok.	
	HC5	I take responsibility for the state of my health after using TikTok.	
	HC6	I’m aware of the state of my health as I go through the day after using TikTok.	
Social Interaction Anxiety (SIA)	SIA1	I feel anxious when talking with people I have just met after using TikTok.	Alkis et al. (2017); X. Zhang et al. (2019)
	SIA2	I feel nervous when I talk with people I do not know very well after using TikTok.	
	SIA3	I feel uneasy while making new friends after using TikTok.	
	SIA4	I feel tense when I meet someone for the first time after using TikTok.	
	SIA5	I am afraid of interacting with others after using TikTok.	
	SIA6	I feel nervous when I have to talk with others about myself after using TikTok.	
Social media fatigue (SMF)	SMF1	I am likely to receive too much information when I am searching for something on TikTok.	Bright et al. (2022)
	SMF2	I am frequently overwhelmed by the amount of information available on TikTok.	
	SMF3	I find that TikTok does not have enough detail to quickly find the information I am looking for.	
	SMF4	The amount of information available on TikTok makes me feel tense.	
	SMF5	When searching for information on TikTok, I frequently just give up because there is too much to deal with.	
Overall user satisfaction (SA)	SA1	How do you feel about your overall experience of this TikTok use?	Sharabati et al. (2022)
	SA2	Are you overall satisfied with the relational benefits brought by TikTok?	
	SA3	Are you overall satisfied with the informational benefits brought by TikTok?	
	SA4	Are you overall satisfied with the enjoyment brought by TikTok?	
	SA5	Are you overall satisfied with the curiosity fulfilment brought by TikTok?	

### Appendix A.2. The EFA Testing Result

	**Dimensions**					
	**IA**	**SA**	**HC**	**PC**	**Time**	**SMF**
SIA3	0.933					
SIA4	0.907					
SIA2	0.905					
SIA6	0.900					
SIA1	0.891					
SIA5	0.866					
SA1		0.873				
SA4		0.871				
SA5		0.866				
SA2		0.839				
SA3		0.831				
HC4			0.853			
HC6			0.853			
HC5			0.770			
HC3			0.766			
HC1			0.694			
HC2			0.689			
PC4				0.928		
PC3				0.922		
PC1				0.851		
PC2				0.837		
Time1					0.867	
Time2					0.861	
Time4					0.853	
Time3					0.819	
SMF2						0.869
SMF1						0.829
SMF4						0.790
SMF5						0.669
Notes: Time: perceived time spent on TikTok; PC: privacy concern; HC: health consciousness; SIA: social interaction anxiety; SMF: social media fatigue.						
